# Assessing and Managing Primary Hyperparathyroidism and Fracture Risk in England: A Survey of Medical Professionals

**DOI:** 10.1210/jendso/bvae225

**Published:** 2025-01-28

**Authors:** Kaiyang Song, Rohit Vijjhalwar, Mo Aye, Alexander N Comninos, Marian Schini, Afroze Abbas, Neil Gittoes, Muhammad Kassim Javaid

**Affiliations:** Medical Sciences Division, University of Oxford, Oxford OX3 9DU, UK; Medical Sciences Division, University of Oxford, Oxford OX3 9DU, UK; Centre for Metabolic Bone Diseases, Hull Royal Infirmary, Hull HU3 2JZ, UK; Endocrine Bone Unit, Imperial College Healthcare NHS Trust, London W2 1NY, UK; Division of Clinical Medicine, School of Medicine and Population Health, University of Sheffield, Sheffield S10 2TN, UK; Leeds Centre of Endocrinology and Diabetes, Leeds Teaching Hospitals NHS Trust, Leeds LS9 7TF, UK; Centre for Endocrinology, Diabetes and Metabolism, Queen Elizabeth Hospital and University of Birmingham, Birmingham B15 2GW, UK; Nuffield Department of Orthopaedics, Rheumatology and Musculoskeletal Sciences, University of Oxford, Oxford OX3 7LD, UK

**Keywords:** hyperparathyroidism, fracture, survey, antiosteoporotic medication

## Abstract

**Purpose:**

To describe diagnostic approaches and management strategies for patients with primary hyperparathyroidism (PHPT) and recent fracture in England.

**Methods:**

We developed a survey based on a patient at high fracture risk and a new diagnosis of probable PHPT. The survey was circulated among 50 secondary care professionals identified by the Society for Endocrinology Calcium and Bone special interest group. Descriptive statistics, combinatorial, and thematic analyses were employed.

**Results:**

In the patient with hyperparathyroidism and a recent fracture, 54% of respondents favoured a 24-hour urinary calcium: creatinine clearance ratio, with 85% opting to do so after correcting vitamin D levels. Thirty-two percent (16/50) preferred the spot urinary calcium:creatinine clearance ratio, as a random test (56%, n = 9/16). Ninety-six percent of the respondents agreed they would include a fracture risk assessment in their management plan. Eighty-five percent of the respondents selected dual-energy X-ray absorptiometry scans of the lumbar spine, total hip, and femoral neck as the most popular choice. Before initiating antiosteoporotic medications (AOMs), 94% of the respondents preferred correcting vitamin D levels with diverse regimens. IV zoledronate acid was the preferred AOM, and 58% (n = 29/50) supported cinacalcet usage if the patient was ineligible for parathyroid surgery, while 26% (n = 13/50) opposed cinacalcet use entirely. No significant correlation was found between status as an endocrinology consultant or working in a tertiary care hospital and these management preferences.

**Main Conclusion:**

This study of National Health Service medical staff identified highly-varied clinical practices in managing PHPT in the setting of high fracture risk, highlighting the need for pragmatic guidelines and wider education.

Primary hyperparathyroidism (PHPT) is a relatively common endocrine disorder characterized by excessive secretion of PTH, leading to dysregulation of calcium homeostasis with hypercalcemia [1]. The condition affects an estimated 1 to 7 per 1000 people in the general population [2, 3]. PHPT typically manifests with nonspecific symptoms such as fatigue, confusion, constipation, muscle weakness, and bone pain, making its diagnosis challenging with an increasing number of cases being diagnosed opportunistically on laboratory testing for other reasons [1, 4, 5]. Furthermore, PHPT is associated with an increased risk of fractures and other skeletal complications, along with kidney stones [6, 7], hypertension [8], and neuropsychiatric disorders such as depression, anxiety, and cognitive impairment [5, 9, 10], emphasizing the importance of accurate diagnosis and effective management strategies [11].

Given the complex nature of PHPT and its diverse clinical presentations, there is considerable variation in how healthcare professionals diagnose and manage the condition. In 2019, in the United Kingdom, the NG132 guideline set by the National Institute of Health and Care Excellence offered recommendations for assessing, diagnosing, and managing PHPT [12]. This guideline highlighted the importance of ruling out familial hypocalciuric hypercalaemia through various possible tests such as 24-hour urinary calcium excretion or random calcium:creatinine ratios [12]. Following diagnosis, a dual-energy X-ray absorptiometry (DXA) scan of the lumbar spine, hip, and distal radius is recommended to evaluate fracture risk. Treatment options include surgical removal of the parathyroid gland (parathyroidectomy) and the usage of oral or IV bisphosphonates to decrease fracture risk.

The Fifth International Workshop on the evaluation and management of primary hyperparathyroidism recommended surgery in the presence of spine fractures or bone mineral density (BMD) ≤ −2.5 at presentation or an incident fracture [13]. For patients inappropriate for surgery, calcimimetics like cinacalcet are recommended [12]. However, it is worth noting that cinacalcet does not significantly alter BMD values or affect fracture risk [14]. Nevertheless, it is now established that there is an imminent fracture risk following an incident fracture; this is significantly higher in the next 2 years after the fracture occurred and identifies patients at very high fracture risk [15, 16]. However, current guidelines do not specifically cover the high imminent fracture risk. With over 520 000 fragility fractures in adults in the UK annually [4] and improved identification and assessment, including testing for hypercalcemia, by Fracture Liaison Services [5], the number of patients at a high imminent fracture risk in the setting of likely PHPT is a growing clinical scenario.

This report aims to describe the diagnostic approaches and management strategies employed by specialist medical professionals in the setting of recent major osteoporotic fractures and probable PHPT in the National Health Service. Understanding the current practices and preferences of specialist medical professionals is crucial to identifying gaps in patient care and priorities for standardization. The findings will inform future clinical guidelines and their revision as well as educational initiatives aimed at improving and standardizing the care of patients with PHPT and high fracture risk.

## Methods

The Society for Endocrinology Endocrine Specialist Network (Sfe ESN) for bone and calcium is a UK-based multidisciplinary network that aims to catalyze cross-institutional and cross-disciplinary collaborative research initiatives and act as a crucial conduit for advice and suggestions from the membership to the Science, Clinical, Nurse, Early Career, Corporate Liaison and Public Engagement Committees. Members of the Sfe ESN for Bone and Calcium highlighted potential variability in the management of adults at high fracture risk and PHPT and initiated the study.

### Study Design

This study utilized a cross-sectional survey design to assess the diagnostic approach, fracture risk determination, and management strategies employed by specialist medical professionals in cases of PHPT. The objectives of the survey were to (1) evaluate adherence to guideline recommendations among medical professionals managing PHPT, (2) identify discrepancies between current practices and established guidelines, and (3) explore physician preferences in areas lacking established consensus.

The Sfe ESN for Bone and Calcium iteratively developed the survey to collect information about the respondent's speciality, grade, and geographic location. A specialist medical professional was defined as any clinician who was either a trainee or consultant or was working within a secondary care speciality. The survey asked management questions for an 80-year-old woman with a recent hip fracture, vitamin D deficiency, and chronic kidney disease 3a with laboratory results consistent with PHPT. Respondents were asked to provide their responses based on their clinical experience and expertise.

The questionnaire consisted of structured and open-ended questions covering various aspects of diagnosis, fracture risk assessment, and management strategies (Supplementary Questionnaire 1) [17].

### Data Collection

Survey participants were healthcare professionals involved in the clinical management of adults at high fracture risk and PHPT. The Sfe SEN for Bone and Calcium used snowball sampling to identify survey participants. Data collection was conducted electronically using an online survey platform. Responses were anonymized to maintain confidentiality and enhance the validity of the study findings. Members of the Sfe for Bone Calcium were encouraged to circulate the survey to colleagues in their region. We compared responses with international recommendations on the diagnosis and management of PHPT where available [1, 13, 18].

### Data Analysis

Quantitative data obtained from the survey were analyzed using descriptive statistics, including frequencies and percentages. Qualitative data from open-ended questions were analyzed thematically and recoded into themes and patterns in the respondents' approaches to the diagnosis and management of PHPT. Combinatorial analyses were used to achieve precise categorization of fracture risk assessment methods and antiosteoporotic medications (AOM) regimens using R packages UpSetR (analysis) [19] and ggupset (visualization). The responses were then compared from consultant endocrinologists vs other respondents and from tertiary hospitals vs other settings where practices showed the greatest variability using chi-squared and Fisher exact tests. Analysis was performed in R Version 2022.07.2 (R Foundation for Statistical Computing, Vienna, Austria).

### Ethics

A General Data Protection Regulation statement was included in the survey, and specific ethics approval was not required.

## Results

### Respondents’ Characteristics

A total of 50 clinicians completed the survey (Table 1). As anticipated, most respondents were consultants working in tertiary hospital settings. While most responses were from London, there were responses from across England. 52% (n = 26) of the respondents had access to specialist metabolic bone clinics and 68% (n = 34) had access to a regional adult bone multidisciplinary network. The predominant group among the respondents from the endocrinology specialty consisted of consultants, representing 81% of the total (n = 22/27).

**Table 1.** Clinician characteristics including grades, specialties, the type of hospital they practice at, and their geographical location

Characteristic	n = 50
**Grade of medical professional (%)**	
Consultant	42 (84)
Trainee	7 (14)
Nurse specialist	1 (2)
**Specialty (%)**	
Endocrinology	27 (54)
Rheumatology	12 (24)
Metabolic medicine	6 (12)
Clinical chemistry	2 (4)
Endocrine surgery	1 (2)
Ear, nose, throat	1 (2)
Fracture prevention	1 (2)
**Hospital type (%)**	
Specialist/tertiary care	36 (72)
District general	14 (28)
**Location of hospital (%)**	
London	21 (42)
Midlands	9 (18)
North East and Yorkshire	9 (18)
South Central	5 (10)
South East	2 (4)
South West	2 (4)
East of England	1 (2)
North West	1 (2)

### Initial Assessment of PHPT

While in the setting of a recent fracture, international guidelines recommend clinicians request a urinary calcium:creatinine ratio to help exclude familial hypercalcaemic hypocalciuria (FHH). [1] However, there is little guidance on precisely how this should be performed. While 54% (n = 27/50) of the respondents would request a 24-hour urine collection, only 85% of those would do so after correcting for vitamin D deficiency, with 61% of the respondents waiting until vitamin D levels exceeded 50 nmol/L. Table 2 summarizes the respondents' assessment of preferred methods for suspected PHPT. Among the subset of respondents who requested a 24-hour urine collection, 85% were consultants (n = 23/27) and 59% were endocrinologists (n = 16/27), with 89% (n = 24/27) employed in tertiary care hospitals.

**Table 2.** Assessment of calcium:creatinine clearance ratio

Options for assessing primary hyperparathyroidism	n = 50
24-hour urinary calcium:creatinine clearance ratio (%)	n = 27 (54)
Calcium:creatinine ratio after correcting vitamin D levels	23 (85)
Calcium:creatinine ratio before correcting vitamin D levels	4 (15)
Spot calcium:creatinine clearance ratio (%)	n = 16 (32)
Random spot calcium:creatinine clearance ratio	9 (56)
Morning spot calcium:creatinine clearance ratio	4 (25)
Fasting spot calcium:creatinine clearance ratio	2 (12)
Unsure when to perform spot calcium:creatinine clearance ratio	1 (6)
Unsure/undecided (%)	n = 7 (14)

Furthermore, there was no consensus on the role of controlling dietary calcium intake during the collection day (40% recommended a range of intake from 500-1200 mg/day). Of the 46% (n = 23/50) of the respondents who did not request a 24-hour urinary calcium:creatinine clearance ratio, 70% (n = 16/23) indicated they would use a spot calcium:creatinine clearance ratio for diagnosing PHPT. Among the 16 respondents preferring a spot test, 56% (n = 9/16) would opt for a random spot test.

### Assessing Fracture Risk in PHPT

**Figure 1.**
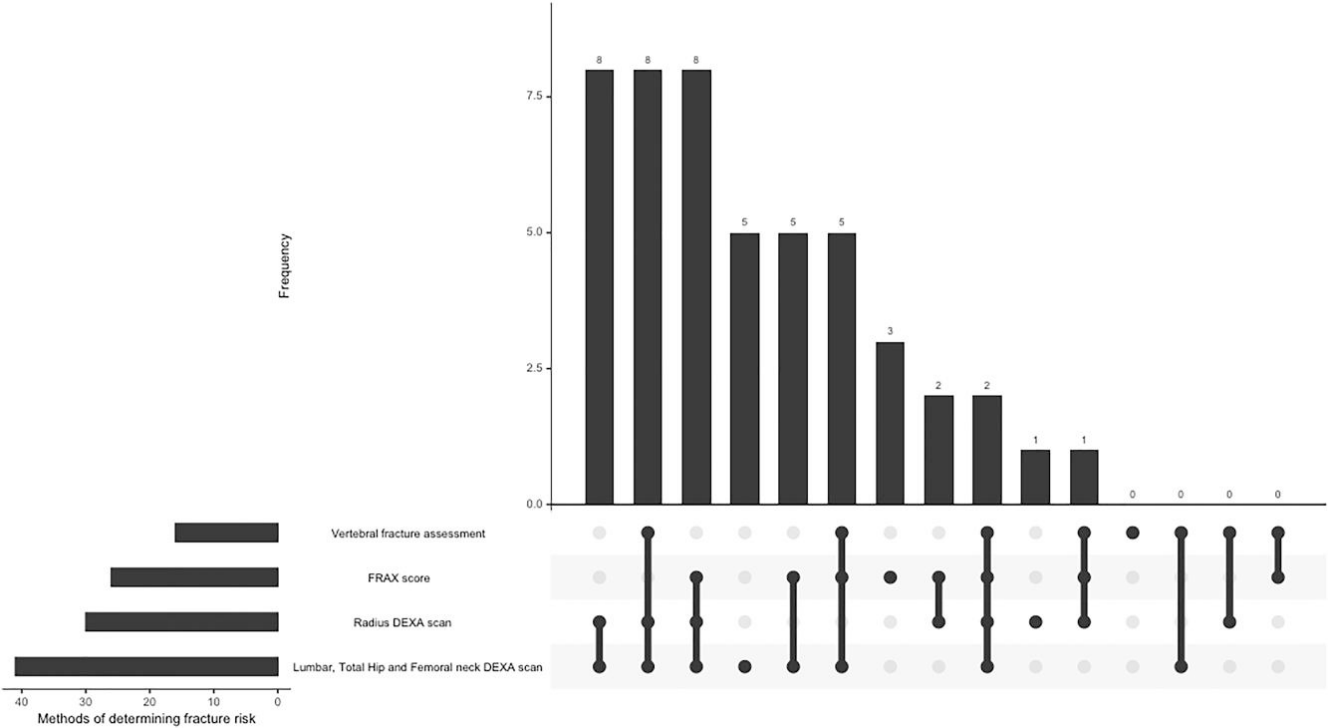
Different combinations of fracture assessment methods for determining fracture risk.

Nearly all respondents recommended fracture risk assessment (n = 48/50, 96%), as shown in Table 3. The majority of respondents (85% of respondents, n = 41/48) indicated they would conduct a fracture risk assessment upon presentation, while 10% (n = 5/48) preferred to wait 3 to 12 months postoperatively following parathyroidectomy. The most prevalent method, chosen by 85% of the respondents, was a DXA scan (of the lumbar spine, total hip, and femoral neck), often in combination with other approaches including vertebral fracture assessment, Fracture Risk Assessment Tool [20], and DXA scan of the radius. Table 3 shows the approaches and their involvement in their overall fracture risk assessment strategy. The most common combination to assess fracture risk was a DXA of the lumbar spine/total hip/femoral neck coupled with a DXA of the radius (17%, n = 8), shown in Fig. 1. Only 2 clinicians would use all 4 tools in their fracture risk assessment (4%).

**Table 3.** Different methods for determining fracture risk

Fracture risk assessment method (multiple choices permitted)	% opted for by respondents (n = 48)
Lumbar, total hip, and femoral neck DXA scan	85.4 (n = 41)
Radius DXA scan	62.5 (n = 30)
FRAX	54.2 (n = 26)
Vertebral fracture assessment	33.3 (n = 16)

Abbreviations: DXA, dual-energy X-ray absorptiometry, FRAX, Fracture Risk Assessment Tool.

### Correcting a Low Vitamin D Before AOM Initiation

The majority of respondents (n = 47/50, 94%) would correct vitamin D levels before initiating oral or IV/subcutaneous AOMs. Of those, more than half of the respondents (n = 27/47, 57%) would correct vitamin D using more than 100 000 units overall in their treatment regimens, whereas 43% of the respondents (n = 20/47) would correct vitamin D using less than 100 000 units overall in their regimens. The most common regimen to correct vitamin D levels was using a weekly dose of vitamin D (24/47, 51% of respondents).

### Use of AOMs and Cinacalcet to Manage Fracture Risk

The majority of the respondents (n = 37 out of 50, 74%) would recommend medication equivalent to other patients with osteoporosis but without primary PHPT. Regarding the optimal timing for commencing AOMs, no consensus was reached: 38% of the respondents (n = 19/50) acknowledged the absence of a standardized approach, indicating variability based on individual patient risk factors. Meanwhile, 30% of the respondents (n = 15/50) expressed a preference for initiating AOMs before parathyroid surgery, while 28% (n = 14/50) favored commencing AOMs post-parathyroid surgery, with some undergoing post-parathyroid surgery DXA scanning (n = 10/50) and others not (n = 2/50). Additionally, there was uncertainty among some of the respondents regarding whether a DXA scan would precede AOM initiation post-parathyroid surgery (n = 2/50).

**Figure 2.**
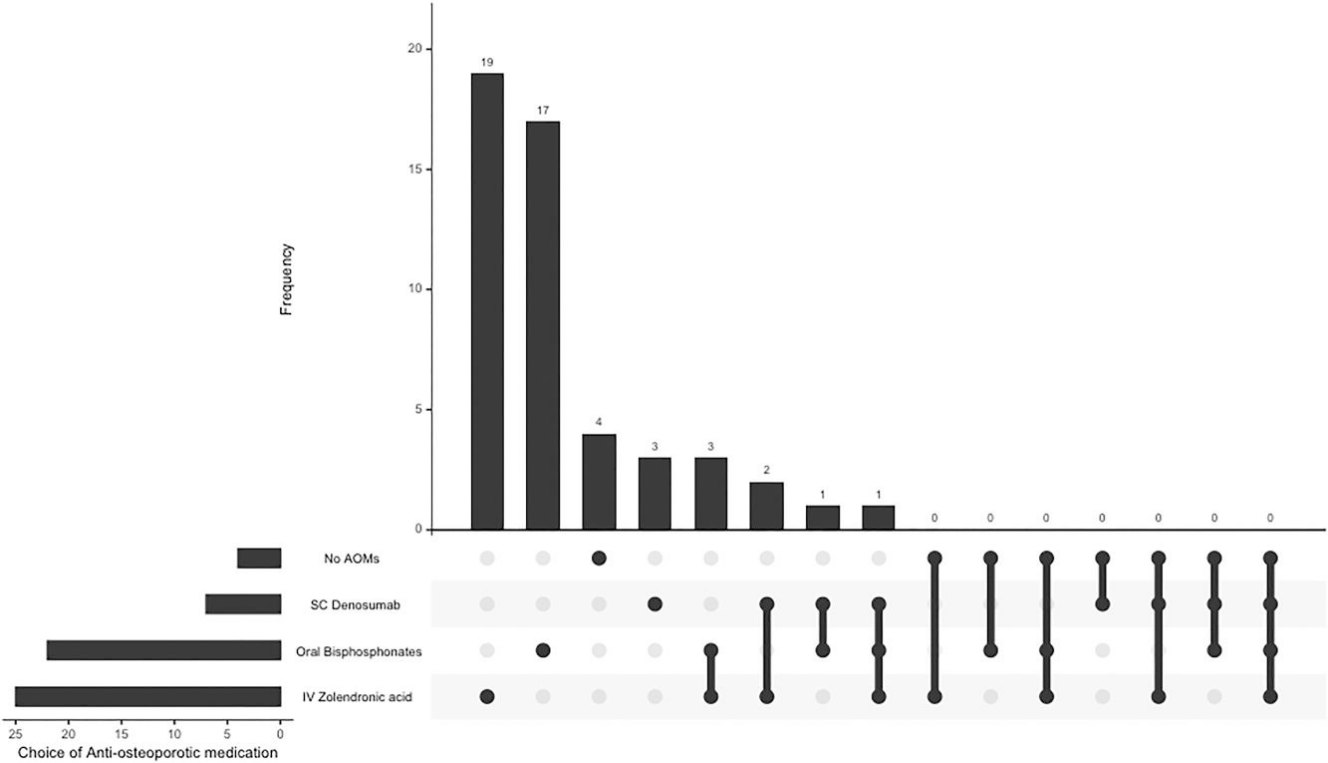
Combinatorial analysis of the antiosteoporotic medications endorsed to be initiated by the respondents.

Table 4 illustrates the preferences for AOM in PHPT, with IV zoledronate as the most favored option, selected by 50% of the respondents (n = 25). Figure 2 shows the predominant combinations of AOMs, with oral bisphosphonates and IV zoledronate forming the most common combination initiated (n = 2/50). The use of cinacalcet to manage fracture risk in PHPT was also variable with most recommending its use in patients ineligible for parathyroid surgery while a quarter felt there was no general role for cinacalcet in this setting (Table 5).

**Table 4.** Different choices for use of AOM in primary hyperparathyroidism

AOM choice	% opted for by respondents (n = 50)
IV zolendronate	50 (n = 25)
Oral bisphosphonates	44 (n = 22)
SC cenosumab	14 (n = 7)
No AOMs	8 (n = 4)

As more than 1 AOM could be selected, the total exceeds 100%.

Abbreviations: AOM, antiresorptive medication, SC, subcutaneous.

**Table 5.** The indications for cinacalcet usage in patients with a recent fracture and primary hyperparathyroidism

Usage of cinacalcet	n = 50
Usage in patients ineligible for parathyroid surgery (%)	29 (58)
No general role for cinacalcet (%)	13 (26)
Only for symptom control (%)	4 (8)
Unsure/undecided (%)	4 (8)

### Endocrinology Consultants Compared to Nonendocrinology Consultants and Tertiary Care Compared to Nontertiary Care Hospitals

No significant correlation was found between being an endocrinology consultant or working in a tertiary care hospital and various decisions, including requests for 24-hour urine collections before or after correcting for vitamin D deficiency, preferences for radius DXA scans in assessing fracture risk, initiation of AOMs before parathyroid surgery, and attitudes toward the general usage of cinacalcet in PHPT patients.

## Discussion

Our data identify a wide variation in the practice of specialized medical professionals in the UK regarding the assessment and management of PHPT in the setting of very high imminent fracture risk. There was marked variability in the role of urinary calcium/creatinine testing, fracture risk assessment, and use of AOMs and cinacalcet, thereby identifying limited awareness and gaps in current national and international guidelines.

A major area of variability is related to the testing of the urinary calcium:creatinine ratio to aid in the exclusion of FHH. Several studies have questioned the value of random spot urine testing compared with 24-hour urine collections, which have lower completion rates. However, these studies are usually in the scenario of urolithiasis/hypercalciuria assessment and not hypocalciuria assessment for FHH. A study of spot urine sampling at 0800 and 1400 showed no difference in the calcium:creatinine ratio compared with 24-hour collections in healthy young adults [21]. While another study demonstrated a similar correlation between these 2 methods [22], in others, there is a poor agreement in studies that may reflect diurnal variation and nonfasting state [19, 23, 24]. In addition, guidelines emphasize the benefit of 24-hour urine collections in PHPT diagnosis due to circadian variability [13]. Other aspects to consider are the availability of historical measurements of serum calcium that show previous normal levels, the effect of fractures increasing 24-hour urine calcium up to 6 weeks postoperatively [25], and the effect of renal impairment (estimated glomerular rate < 60 mL/min/m^2^) on reducing calcium excretion [26]. In addition, vitamin D supplementation may increase urine calcium levels in patients though this rise is not necessarily correlated with serum vitamin D levels, potentially implicating other factors such as dietary intake [27].

The role of bisphosphonates and denosumab in reducing fracture risk in the setting of PHPT is based on improvements in BMD but with no fracture data [28]. While cinacalcet reduces serum calcium, there is no clear effect on fracture risk, bone density at the lumbar spine, total femur, or distal third radius, but there is an increase in resorption and formation of bone markers at 1 year [29] and 5 years [14].

While National Institute of Health and Care Excellence NG132 and international guidelines recommend the use of cinacalcet for patients who are ineligible for or refuse parathyroidectomy, or in cases of refractory PHPT despite parathyroidectomy [1], this survey revealed mixed opinions among the respondents. Some endorsed its use in specific patient populations, while others expressed skepticism regarding its utility, even with established guidelines. This variation in opinion is important, given that a recent meta-analysis demonstrated that cinacalcet could normalize hypercalcemia in 90% of patients with PHPT, significantly reduce PTH levels, and increase serum phosphate levels [30].

Furthermore, the variability in the optimal timing for initiating AOMs highlights the heterogeneity in clinical decision-making. This is particularly relevant given the imminent risk of fracture, time to benefit from AOMs [31], and potential time to wait for surgical excision from initial referral in the National Health Service. While NG132 guidelines provide general recommendations for AOM initiation, the survey findings suggest variations in practice, reflecting the lack of evidence for the superiority of 1 approach over another. One concern is how AOMs preparathyroidectomy may increase the risk of postoperative hypocalcemia [32], although this is not a consistent finding in the literature.

However, this study has notable limitations. The survey focused on a specific case (hypercalcemic PHPT in the context of a recent fracture, chronic kidney disease, and vitamin D deficiency), which may not reflect the practitioner's general practice. Consecutively, the responses might represent their approach to this particular case rather than their usual clinical practice. The inclusion of a wide range of specialities, such as endocrinology and rheumatology, and representation across specialist and nonspecialist settings throughout England provided a broad perspective but also introduced variability in responses. Nearly half (48%) of participants lacked access to specialized metabolic bone clinics, potentially influencing their management strategies. The relatively small sample size of 50 respondents limits the generalizability of the findings. Further work should aim for a larger and more homogenous sample to provide more robust insights. Additionally, respondents reported clinical practice preferences rather than patient-level data describing actual care received. Future work may develop patient-level indicators that can be used to measure actual care received by patients. The study did not capture whether the respondent choices were influenced by clinical preferences or local service availability. In terms of sequencing of management, we did not test the ordering of correction of vitamin D deficiency vs initiation of cinacalcet; this could be tested in future surveys. The case was specifically chosen based on a relatively common clinical intersection, given the epidemiology of both osteoporosis and PHPT, to inform the need for a specific best practice recommendation set. Future surveys may address other clinical scenarios. For example, to ensure the survey focused on clinical consideration related to the intersection between high fracture risk and primary hyperparathyroidism, we did not test the effect of chronic kidney disease on the assessment methodology [33] or AOM eligibility [34]; future studies may elicit responses for assessment and management across different stages of chronic kidney disease and other parameters.

The issue of 24-hour urinary calcium:creatinine clearance ratio collection was also identified as a limitation. While the survey aimed to reflect current variability in clinical practice, it did not account for practical challenges, such as cognitive impairment in elderly patients, which might influence the feasibility of 24-hour urine collection. Future surveys could also include practical issues around 24-hour collections, such as cognitive impairment. Finally, the study did not assess respondents' awareness of national and international guidelines, which is crucial for planning interventions to reduce variability in care delivery.

Our study highlights the absence of robust evidence to guide the management of PHPT, despite established guidelines. This underscores the need for further research to inform personalized care and careful evaluation of the cost-effectiveness of each test and treatment in relation to individual patient scenarios and care objectives. The observed variability in clinical management in clinical practices for managing high fracture risk in PHPT patients indicates a pressing need to establish consensus-based best practice recommendations, enhance the sharing of existing evidence, and identify critical areas of future research. Addressing these issues will be crucial to improving care for this vulnerable patient group.

## Data Availability

Some or all datasets generated during and/or analyzed during the current study are not publicly available but are available from the corresponding author on reasonable request.

## References

[1] Walker MD, Silverberg SJ. Primary hyperparathyroidism. Nat Rev Endocrinol. 2018;14(2):115‐125. 28885621 10.1038/nrendo.2017.104 PMC6037987

[2] Yeh MW, Ituarte PHG, Zhou HC, et al Incidence and prevalence of primary hyperparathyroidism in a racially mixed population. J Clin Endocrinol Metab. 2013;98(3):1122‐1129. 23418315 10.1210/jc.2012-4022 PMC3590475

[3] Soto-Pedre E, Newey PJ, Leese GP. Stable incidence and increasing prevalence of primary hyperparathyroidism in a population-based study in Scotland. J Clin Endocrinol Metab. 2023;108(10):e1117‐e1124. 37022975 10.1210/clinem/dgad201 PMC10505547

[4] Silverberg SJ, Clarke BL, Peacock M, et al Current issues in the presentation of asymptomatic primary hyperparathyroidism: proceedings of the fourth international workshop. J Clin Endocrinol Metab [Internet]. 2014;99(10):3580‐3594. 25162667 10.1210/jc.2014-1415 PMC5393491

[5] Cope O . The story of hyperparathyroidism at the Massachusetts general hospital. N Engl J Med. 1966;274(21):1174‐1182. 5327350 10.1056/NEJM196605262742105

[6] Silverberg SJ, Shane E, Jacobs TP, Siris E, Bilezikian JP. A 10-year prospective study of primary hyperparathyroidism with or without parathyroid surgery. N Engl J Med [Internet]. 1999;341(17):1249‐1255. 10528034 10.1056/NEJM199910213411701

[7] Rejnmark L, Vestergaard P, Mosekilde L. Nephrolithiasis and renal calcifications in primary hyperparathyroidism. J Clin Endocrinol Metab [Internet]. 2011;96(8):2377‐2385. 21646371 10.1210/jc.2011-0569

[8] Walker MD, Silverberg SJ. Cardiovascular aspects of primary hyperparathyroidism. J Endocrinol Invest [Internet]. 2008;31(10):925. 19092300 10.1007/BF03346443 PMC6056175

[9] Albright F, Aub J, Medical WBJ of the A, 1934 undefined. Hyperparathyroidism: a common and polymorphic condition as illustrated by seventeen proved cases from one clinic. jamanetwork.com [Internet]. [cited 2024 Jul 18]; Available from: https://jamanetwork.com/journals/jama/article-abstract/249530

[10] Walker MD, Silverberg SJ. Parathyroidectomy in asymptomatic primary hyperparathyroidism: improves “bones” but not “psychic moans.”. J Clin Endocrinol Metab. 2007;92(5):1613‐1615. 17483374 10.1210/jc.2007-0551

[11] Kanis JA, Harvey NC, Liu E, et al Primary hyperparathyroidism and fracture probability. Osteoporos Int. 2023;34(3):489‐499. 36525071 10.1007/s00198-022-06629-y

[12] Hyperparathyroidism (primary): diagnosis, assessment and initial management (2019) NICE guideline NG132. 31194309

[13] Bilezikian JP, Khan AA, Silverberg SJ, et al Evaluation and management of primary hyperparathyroidism: summary statement and guidelines from the fifth international workshop. J Bone Miner Res. 2022;37(11):2293‐2314. 36245251 10.1002/jbmr.4677

[14] Peacock M, Bolognese MA, Borofsky M, et al Cinacalcet treatment of primary hyperparathyroidism: biochemical and bone densitometric outcomes in a five-year study. J Clin Endocrinol Metab [Internet]. 2009;94(12):4860‐4867. 19837909 10.1210/jc.2009-1472

[15] Wong RMY, Wong PY, Liu C, et al The imminent risk of a fracture-existing worldwide data: a systematic review and meta-analysis. Osteoporos Int [Internet]. 2022;33(12):2453‐2466. 35776148 10.1007/s00198-022-06473-0

[16] Kanis JA, Johansson H, Odén A, et al Characteristics of recurrent fractures. Osteoporos Int [Internet]. 2018;29(8):1747‐1757. 29947869 10.1007/s00198-018-4502-0 PMC6076437

[17] Vijjhalwar R. Supplementary data. Mendeley Data [Internet]. 2024;V1. Available from: https://data.mendeley.com/datasets/ng8sm3fwcd/1

[18] Jawaid I, Rajesh S. Hyperparathyroidism (primary) NICE guideline (NG132): diagnosis, assessment, and initial management. The British Journal of General Practice [Internet]. 2020;70(696):362. 32586826 10.3399/bjgp20X710717 PMC7319687

[19] Hong YH, Dublin N, Razack AH, Mohd MA, Husain R. Twenty-four hour and spot urine metabolic evaluations: correlations versus agreements. Urology [Internet]. 2010;75(6):1294‐1298. 19914693 10.1016/j.urology.2009.08.061

[20] Kanis JA, Johnell O, Oden A, Johansson H, McCloskey E. FRAX^TM^ and the assessment of fracture probability in men and women from the UK. Osteoporosis International [Internet]. 2008;19(4):385. 18292978 10.1007/s00198-007-0543-5 PMC2267485

[21] Topal C, Algun E, Sayarlioglu H, et al Diurnal rhythm of urinary calcium excretion in adults. Ren Fail. 2008;30(5):499‐501. 18569929 10.1080/08860220802060471

[22] Gökçe Ç, Gökçe Ö, Baydinç C, et al Use of random urine samples to estimate total urinary calcium and phosphate excretion. Arch Intern Med [Internet]. 1991;151(8):1587‐1588. 1872663 10.1001/archinte.1991.00400080083015

[23] Strohmaier WL, Hoelz KJ, Bichler KH. Spot urine samples for the metabolic evaluation of urolithiasis patients. Eur Urol. 1997;32(3):294‐300. 9358216

[24] Ferraro PM, Lopez F, Petrarulo M, et al Estimating 24-hour urinary excretion using spot urine measurements in kidney stone formers. Nephrology Dialysis Transplantation [Internet]. 2022;37(11):2171. 35146503 10.1093/ndt/gfab306 PMC9585473

[25] Wang J, Zheng X, Zhang L, et al The variation in urinary calcium levels in adult patients with fracture and surgical intervention. J Orthop Surg Res [Internet]. 2017;12(1):1‐7. 28810891 10.1186/s13018-017-0624-x PMC5558773

[26] Cirillo M, Bilancio G, Cavallo P, et al Reduced kidney function and relative hypocalciuria—observational, cross-sectional, population-based data. J Clin Med [Internet]. 2020;9(12):1‐15. 10.3390/jcm9124133 PMC7767498 33371520

[27] Taheri M, Tavasoli S, Shokrzadeh F, Amiri FB, Basiri A. Effect of vitamin D supplementation on 24-hour urine calcium in patients with calcium urolithiasis and vitamin D deficiency. International Brazilian Journal of Urology : official journal of the Brazilian Society of Urology [Internet]. 2019;45(2):340. 10.1590/S1677-5538.IBJU.2018.0522 PMC6541149 30735332

[28] Ye Z, Silverberg SJ, Sreekanta A, et al The efficacy and safety of medical and surgical therapy in patients with primary hyperparathyroidism: a systematic review and meta-analysis of randomized controlled trials. J Bone Miner Res [Internet]. 2022;37(11):2351‐2372. 36053960 10.1002/jbmr.4685

[29] Peacock M, Bilezikian JP, Klassen PS, Guo MD, Turner SA, Shoback D. Cinacalcet hydrochloride maintains long-term normocalcemia in patients with primary hyperparathyroidism. J Clin Endocrinol Metab [Internet]. 2005;90(1):135‐141. 15522938 10.1210/jc.2004-0842

[30] Ng CH, Chin YH, Tan MHQ, et al Cinacalcet and primary hyperparathyroidism: systematic review and meta regression. Endocr Connect [Internet]. 2020;9(7):724. 32621588 10.1530/EC-20-0221 PMC7424342

[31] Deardorff WJ, Cenzer I, Nguyen B, Lee SJ. Time to benefit of bisphosphonate therapy for the prevention of fractures among postmenopausal women with osteoporosis: a meta-analysis of randomized clinical trials. JAMA Intern Med [Internet]. 2022;182(1):1. 34807231 10.1001/jamainternmed.2021.6745 PMC8609461

[32] Corsello SM, Paragliola RM, Locantore P, et al Post-surgery severe hypocalcemia in primary hyperparathyroidism preoperatively treated with zoledronic acid. Hormones [Internet]. 2010;9(4):338‐342. 21112866 10.14310/horm.2002.1286

[33] Liu J, Tio MC, Verma A, et al Determinants and outcomes associated with urinary calcium excretion in chronic kidney disease. J Clin Endocrinol Metab [Internet]. 2022;107(1):E281‐E292. 34390334 10.1210/clinem/dgab574 PMC8684460

[34] Evenepoel P, Cunningham J, Ferrari S, et al Diagnosis and management of osteoporosis in chronic kidney disease stages 4 to 5D: a call for a shift from nihilism to pragmatism. Osteoporos Int [Internet]. 2021;32(12):2397‐2405. 34129059 10.1007/s00198-021-05975-7

